# Equity Lens on Canada’s COVID-19 Response: Review of the Literature

**DOI:** 10.34172/ijhpm.2024.8132

**Published:** 2024-05-07

**Authors:** Muhammad Haaris Tiwana, Julia Smith, Megan Kirby, Simran Purewal

**Affiliations:** ^1^Faculty of Health Sciences, Simon Fraser University, Burnaby, BC, Canada.; ^2^Department of History, Faculty of Liberal Arts & Professional Studies, York University, Toronto, ON, Canada.

**Keywords:** Canada, COVID-19, Equity-Focus, Pandemic Preparedness, Policy Planning, Vulnerable Populations

## Abstract

**Background:** A growing literature has documented how the secondary effects of the COVID-19 pandemic have compounded socioeconomic vulnerabilities already present in society, particularly across social categories such as gender, race, class, and socioeconomic status. Such effects demonstrate how pandemic response policies act as structural determinants of health to influence not only direct health outcomes but also intermediary outcomes, such as access to education or income.

**Methods:** This review aims to scope research that analyzes pandemic response policies in Canada from an equity perspective, to identify common themes, recommendations, and gaps.

**Results:** Fourteen studies were thematically analyzed, the majority being qualitative policy document analysis, applying critical frameworks and focused on effects on select priority populations. Analysis of economic and labour policies indicates a lack of consideration for the specific needs of priority populations, and those engaged in precarious, informal, and essential labour. Analysis of social policies illustrate the wide-ranging effects of school and service closures, particularly on women and children. Furthermore, these policies lacked consideration of populations marginalized during the pandemic, include older adults and their caregivers, as well as lack of consideration of the diversity of Indigenous communities. Recommendations proposed in this review call for developing policy responses that address persistent social and economic inequities, pandemic response policies tailored to the needs of priority populations and more meaningful consultation during policy development.

**Conclusion:** The limited number of studies suggests there is still much scope for research recognizing policies as structural determinants of health inequities, including research which takes an intersectional approach.

## Background

 In March 2020, shortly after the World Health Organization (WHO) declared a global Public Health Emergency of International Concern, the Canadian government enacted policies and a series of measures to contain the spread of COVID-19.^1^ These included the closures of schools, childcare centers, community services, most non-essential businesses, and restrictions on cross-border travel and gathering.^2^ Additionally, health and social services were either closed, reduced, or significantly modified due to the prevailing guidelines.^2^

**Figure 1:**
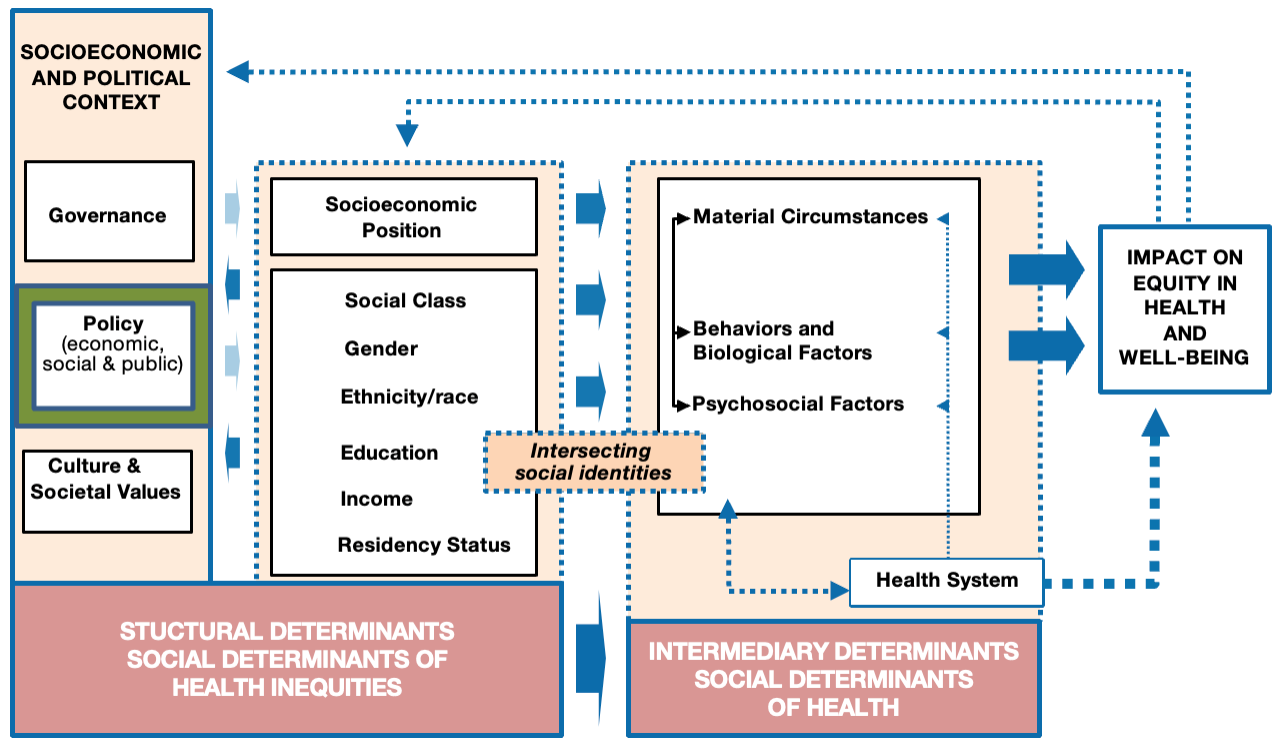
Social Determinant of Health Model (Adapted From “WHO Social Determinants of Health Framework”).

 The direct health impacts of COVID-19, which resulted in approximately 132 000 hospitalizations and over 35 000 deaths to date in Canada, are not the pandemic’s only implications.^3,4^ The secondary effects include those that resulted from measures aiming to mitigate the direct effects.^5^ The epidemic brought to light long-standing structural social and health inequities, including unstable and unfavourable working circumstances, widening economic gaps, and biased political institutions.^1^ Although COVID-19 has proven to be a highly contagious disease, possibly infecting over 50% of all Canadians, for some, lifestyle, employment, and income privileges provided auxiliary layers of protection.^6^ The extant literature has documented how the secondary effects of the pandemic have compounded socioeconomic vulnerabilities already present in society, particularly across social categories such as gender, race, class, socioeconomic status, and other factors.^7-9^ These effects illustrate how pandemic response policies act as structural determinants of health, alongside biological determinants such as the COVID-19 virus, to influence not only direct health outcomes but also intermediary outcomes, such as access to education or income^10^ (Figure 1). Equity-based analysis of such policies can enhance our understanding of why priority populations disproportionately experienced secondary effects of the pandemic and inform future responses to prevent such impacts. Priority populations are those population groups most at risk of the negative effects of the pandemic (ie, most likely to experience inequities) including but not limited to women, ethnic and racialized persons, people with disabilities, immigrants, older adults, and 2SLGBTQ+ (Two-Spirit, Lesbian, Gay, Bisexual, Transgender, Queer, and other identities) identifying peoples.^11^

 This review aims to scope research that analyzes pandemic response policies in Canada from an equity perspective. We define a health equity approach as one focused on societal efforts to address avoidable inequalities, historical and contemporary injustices, and the elimination of health and healthcare inequities.^12^ Through analysis of peer reviewed literature, we explore how research has applied a health equity lens to assess policy responses, and what common themes, recommendations and gaps emerge.

 Notably, the focus of this review is on policy analysis and assessments focused on priority populations inferred from the literature, as opposed to population-level impacts and outcomes. That is research that has policy (defined here as formal actions taken by governments) as its central focus, as opposed to producing evidence to inform policy. While there is the intent that such analysis will in turn to contribute to the future policy development, the research focus is on the policy (green box in figure one above), as opposed to population groups and health behaviours (the other two boxes in figure one above).

 Canada is a particularly interesting country for equity-based policy analysis as the government has made notable commitments to an equity-based response. In 2016, the government released the Gender-based Analysis Plus (GBA Plus) action plan which underscored strengthening networks to assess systemic inequalities and enhancing GBA Plus training for officials.^13^ With the passing of the Canadian Gender Budgeting Act, it mandated the inclusion of GBA Plus for all new annual budget measures or tax expenditures.^14^ The government’s GBA Plus is an intersectional analytical tool used to assess and develop inclusive policy and programming that goes beyond biological and gendered differences but also consider other factors such as age, disability, ethnicity, geography, religion etc. The federal government has committed to mainstreaming GBA Plus across policy spheres, including it in assessments of COVID-19 policies, and has described its pandemic response as “feminist.”^15^ Despite these stated intentions, research has documented how priority populations have been disproportionately affected by the secondary effects of the pandemic. This raises questions about the implementation gaps—the difference between policy intent and outcomes—to inform more effective equity-based policy in the future.

## Methods

 We used an abbreviated version of the PRISMA-ScR (Preferred Reporting Items for Systematic reviews and Meta-Analyses extension for Scoping Reviews) using the 20-point checklist.^16^ The objective was to scope literature analyzing how COVID-19 response policies mitigated and exacerbated health and social inequities among priority populations, with priority populations defined as those population groups most at risk of socially produced health inequities.^11^

###  Search and Screening Strategy

**Figure 2:**
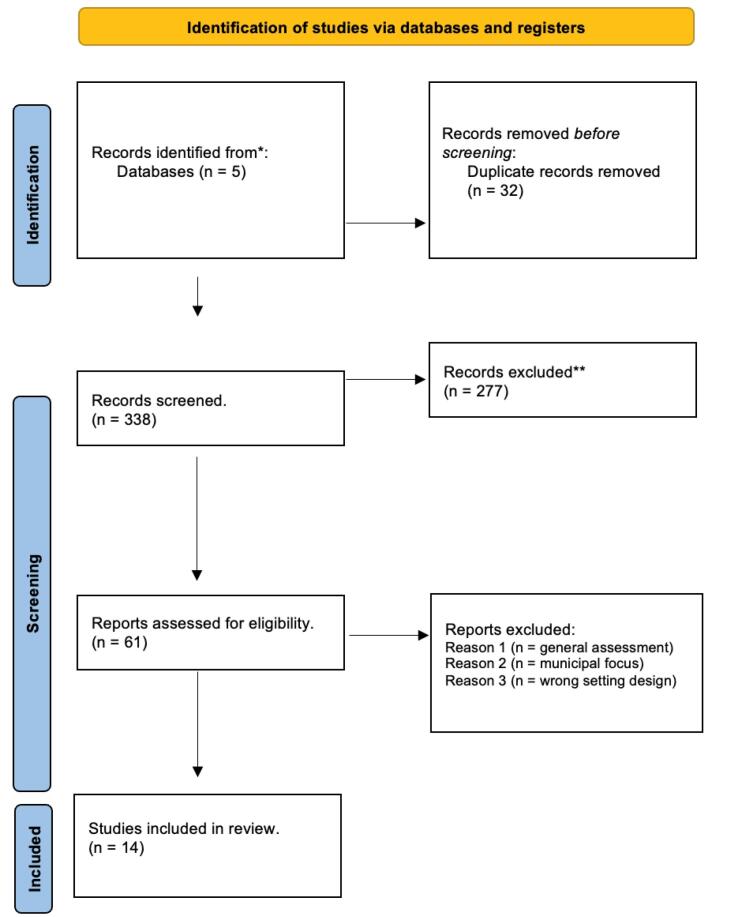
Flow Chart for Identification of Studies.

 We searched and cross-referenced results from five databases (Scopus, PubMed, CINAHL, JSTOR, and Web of Science), using Boolean operators (See Supplementary file 1 for search terms). Furthermore, we reviewed the websites of relevant civil society organizations and research centres that focus on policy and social determinants. The inclusion criteria for our study were that the literature should be: (1) Canadian and COVID-19 focus (January 2020 till May 2023), (2) focus on of federal or nation-wide (ie, across multiple provinces/contexts) policies (defined as a formal action taken by the government) related to the pandemic, (3) include an equity, gender, or intersectional perspective for one or more priority populations, (4) peer-reviewed. Excluded literature from our study were those focused on municipal level policies or a single province, and experimental design studies. Notably, as our focus is on the policy literature, while our search terms include priority populations, those articles that documented effects on priority populations but did not include an element of policy analysis were excluded (for example an article that documents decreased income among a certain group but does not discuss economic policy in a meaningful way would be excluded). Literature was considered for inclusion if it either addressed the overall impact of policies on priority populations or evaluated a specific policy and its relational impact on the selected population. Similarly, due to our equity focus, general assessments of Canada’s pandemic response that did not include mention of priority populations or equity considerations were also not considered (Figure 2).

 Two authors were involved in the literature search and screening. One author conducted the search through the databases and imported identified literature. They identified a total of 370 articles and after importing to Covidence, 32 duplicates were removed. The two reviewers proceeded to independently screen titles and abstracts through Covidence, collaboratively reviewing and resolving conflicts, removing another 277 articles, and then another 61 following full-text screening.

###  Data Extraction

 For the remaining 14 studies, employing a content analysis framework, we extracted the following data: title, authors, publication date, aim of study, method, framework/theory, population of focus, timeline of analysis, background of the policy or measure being assessed, findings regarding the impact of the policy on priority population(s), and policy recommendations. Based on these findings and discussions among the authors, we organized the data according to policy sectors and then sub-themes.

###  Study Limitations

 Inclusion of only peer-reviewed literature did mitigate the risk of bias to a degree.^17^ However, the exclusion of grey literature disregards learnings from civil society reports and media articles that could have proved useful for policy insights regarding priority populations. Including articles available only in English also reduced the breadth of literature, especially from Quebec.

## Results

###  Descriptive Results

 The literature review included a total of 14 articles thematically screened that assessed Canada’s pandemic response policy from an equity perspective or with a focus on effects on priority populations. Most identified articles used a qualitative approach, with data collected through policy document analysis (n = 7) and interviews with stakeholders (n = 2), including two comparative countries analyses. Some studies employed a mixed-methods approach (n = 3), combining qualitative data with quantitative surveys to provide a multidimensional analysis of policy outcomes.

 Critical policy analysis was the most common framework applied in the reviewed studies (6 studies in total), used to analyze power imbalances in policy decision-making and examination of the implications of these imbalances on priority populations.^18-23^ A security resilience framework was used to understand the interdependence and complexity of social, economic, and environmental factors of a population group.^24^ Lee et al^25^ applied the concept of necropolitics to argue that policies rooted in structural violence and racial capitalism led to preventable suffering and death. The social determinant of health model was used to identify and assess social determinants and their impacts on health and social supports.^26^ Similarly, an intersectional approach was used to understand the complex and interrelated ways in which different social identities intersect to shape experiences of health and social well-being during the pandemic.^27^ Koebel et al^28^ applied the Efficiency, Equity and Voice framework to compare income support programs during the COVID-19 crisis. Also conducting a comparative analysis, Katz and colleagues^29^ used an open systems approach to identify the impact of the political, legal, socioeconomic, and cultural context and assess limitations.

 All the studies acknowledged and elaborated on the pre-existing vulnerabilities and systemic barriers that priority populations have faced and how pandemic policies further exacerbated these inequities. Table lists which priority populations were identified in the literature as experiencing secondary effects due to pandemic policies. Four articles (Pin et al, Doucet et al, Smith et al, Combden et al) describe how intersecting identities, such as race, gender, and class acted as compounding factors to amplify social impacts during the pandemic.^20,23,27,30^ Among them, two define intersectionality and discuss how this lens influenced their methodology. Pin et al^20^ explains intersectionality as an analytical tool, and a praxis that can help to reveal and respond to societal injustices resulting from complex inequalities. Smith et al^27^ define it as the multiple ways in which oppressive systems overlap, recognizing injustices are based not only on gender, but also on race, ethnicity, sexuality, economic background, (dis)ability, geography, and religion, and other sources of discrimination and subordination. The remaining articles focus on a single population without considerations of intersecting social identities.

**Table:** Timeline and Methodologies Adopted to Analyse COVID-19 Policy in Included Studies

**Article **	**Timeline of Analysis **	**Methodologies**	**Priority Population**	**Type of Policy**
Abu Alrob and Shields^24^	Mar 2020–Feb 2021	Mixed methods	Migrants	Economic & Labour
Esses et al^18^	Mar 2020–Feb 2021	Qualitative	Migrants	Social
Lee et al^25^	Mar 2020–Feb 2021	Qualitative	Migrants	Economic & Labour
Beland et al^22^	Mar 2020–May 2021	Qualitative	Low socioeconomic status	Economic & Labour
Pin et al^20^	Mar 2020–Aug 2020	Qualitative	Low socioeconomic status	Economic & Labour
Koebel et al^28^	Mar 2020–Oct 2020	Qualitative	Low socioeconomic status	Economic & Labour
Doucet et al^23^	Mar 2020–Aug 2020	Mixed methods	Women	Social
Johnston et al^31^	Apr 2020–Jul 2020	Mixed methods	Women	Social
Katz et al^29^	Mar 2020–May 2020	Qualitative	Children	Social
Stall et al^21^	Mar 2020–Jul 2020	Qualitative	Older adults	Social
Spence et al^22^	Mar 2020–May 2020	Qualitative	Indigenous	Social
Smith et al^27^	Mar 2020–Jul 2020	Qualitative	Women	Economic & Labour; Social
Combden et al^30^	Feb 2020–Jul 2020	Qualitative	Low socioeconomic status, older adults, 2SLGBTQ+, and racialized persons	Economic & Labour
Ruckert et al ^26^	Mar 2020–Oct 2020	Qualitative	Indigenous, women, people with disabilities, racialized persons	Social

Abbreviation: 2SLGBTQ+, Two-Spirit, Lesbian, Gay, Bisexual, Transgender, Queer, and other identities.

###  Economic and Labour Policy 

 Studies that focus on economic policy highlight a lack of consideration of the specific needs of priority populations and those engaged in precarious, informal, and essential labour. Beland et al^19^ critique the government’s income support and economic relief initiatives as they implemented a blanket strategy based on income levels, rather than concentrating on those with the greatest need. They find that while the federal response decreased income inequality, as measured by disposable household income, the all-encompassing nature of these policies were unsustainable and potentially over-compensatory, as they failed to prioritize specific deserving groups, particularly those with low socioeconomic status. Similarly, Pin et al^20^ find that although the federal government’s income support programs had a notable impact, leading to an 18% rise in income for low-income groups and a 2% reduction in the income gap between the lowest and highest earners in 2020, the programs disregarded crucial social and economic factors, such as addressing vulnerability in precarious employment conditions and the unpaid care economy, which exacerbated pre-existing inequities. Individuals with disabilities, for example, faced additional barriers to accessing government supports and services, due to, for instance, lack of transportation either through reduced service or unable to rely on others because of lockdowns.^26^

 Adopting a gender-specific lens, Smith and collegues^27^ find that though the government’s GBA Plus assessment acknowledged the uneven job losses incurred by women, policies did not prioritize their economic security. While the expansion of federal economic relief policies eligibility criteria and thresholds aided to support women who were concentrated in low-paid and temporary occupations, without targeted measures, many women faced barriers in accessing policies such as the Canada Emergency Response Benefit (CERB). A noted gap in supports was that income support did not ensure access to necessities such as food and PPE. While increased funding to non-profits did aid some populations, this came in the form of charity as opposed to public programs that likely would have reached a larger group. Combden et al^30^ review of pandemic response policies found similar effects, including significant effects on the financial security of 2SLGTBQ+ populations, with 53% of 2SLGTBQ+ households impacted by reduced hours and layoffs, compared to 39% of Canadian households.

 Numerous studies note that the eligibility criteria for economic assistance programs excluded many of those most in need of economic relief such racialized and Indigenous persons, or people of low-income.^18-20,24-26,28^ Positioning economic relief programs, such as CERB, as taxable benefits meant only those who had filed taxes the previous year were eligible, with all workers who made less than CAD $5000 considered ineligible. This criterion restricted access for priority populations engaged in precarious or informal labour. For example, Smith et al^27^ find that women who were most financially vulnerable, such as newcomers, were unable to access many of the programs. Koebel et al^28^ similarly underscored how the focus on formal labour and employment structures perpetuated pre-existing structural vulnerabilities of certain groups including people with disabilities, people with low socioeconomic status, racialized groups, and single parents that tend to be involved in precarious work.

 Research further highlights that restricting benefits to people laid off due to the COVID-19 economic downturn limited options for those who did not feel safe at work. Pin et al^20^ argue that excluding individuals engaged in essential jobs such as working in long-term care homes, food production, and hospital support exposed them to greater risks of exposure. These workers were less likely to be employed in occupations that can be performed from home, most likely to be in low-paid jobs, and worked significantly more hours during COVID-19 restrictions than those in top quintile. Despite public health guidelines, the privilege of physically distancing at work and working from home was not available for many priority populations concentrated in low-income jobs. For example, racialized people are overrepresented among essential service workers a group required to work outside the home. Meanwhile, employers benefited from the Canada Emergency Wage Subsidy, which provided renumeration to employers to reduce layoffs. This resulted in a power imbalance, as workers faced the compounding factors of lack of alternative income sources and restricted access to government’s economic relief programs. Koebel et al^28^ further note that ambiguity regarding access to employment insurance or CERB may have contributed to the rise in unsafe working conditions and mistreatment for employees in precarious work.

 A few studies focus particularly on migrant labourers. Lee at al^25^ underscore the pre-existing restrictive policies and conditions of migrants and temporary workers under the Seasonal Agricultural Worker program. This study highlighted the adoption of practices during COVID-19 that further limited workers’ right to nourishment, physical and mental integrity. Their movement outside of the workplace was restricted, communication with friends and family limited, and even procuring necessities was a challenge. Furthermore, private security was assigned to watch the workers and prevented them from engaging socially or entering the local community. As workers’ legal residency status is dependent on employer, the pandemic policies made the position of these workers even more precarious and vulnerable to abuse. Abu Alrob and Shields emphasized how outbreaks in migrant housing can be attributed to substandard living conditions.^24^ The health risks associated with the bus and airport transfers, enclosed camp spaces, and refusal of visas on humanitarian grounds put migrant labourers in precarious and unsafe living conditions. Many migrants experienced delays in family reunification and sponsorship applications, leaving them separated and in limbo. Additionally, the border closures and international travel measures during the pandemic resulted in the highest number of deportations of refugees since 2015, and some asylum seekers were stranded at borders, where they faced an increased risk of persecution.

###  Social Policy 

 Many studies discuss how the decision to close schools and childcare (provincial-level policies following federal guidance) during the first few months of the pandemic affected parents, particularly mothers. Johnson et al,^31^ for example, report a 37% additional increase in childcare obligations for both men and women, with 2.5 times more hours per week for women. Prentice reported that, “When children are in school, this is when mothers’ labour force participation rate jumps to its highest” while employment recovery during the first year of COVID-19 has been slowest for mothers with school-aged children.^32^ Doucet and colleagues analyse the tripartite parental leave system and the differing leave entitlements and benefits across the country.^23^ They find that the impacts of the pandemic meant that parents could not meet the 600-hour insurable threshold to qualify for maternity and paternal leave benefits, and that the early months of the pandemic more adversely affected mother’s employment than that of fathers. Smith et al^27^ note the federal government responded to increased unpaid care work through cash transfer programs, such as the childcare benefit, and by funding civil society organizations supporting families with specific needs. However, they note that these policies did not explicitly recognize the gendered nature of, or notably reduce the burden of, unpaid care work. Furthermore, the loss of access to social services more adversely impacted newcomer families and families with children with disabilities. Consequently, Smith et al^27^ suggest policy choices that increased care burdens during the initial months of the pandemic may have long-term negative effects on women’s career trajectories and mental health. These findings are further explored in research by Johnston et al who, through a comparative study, document the significant impact of increased unpaid care burdens on mental health among Canadian women.^31^

 During pandemic-related school closures, school boards attempted to support individual students, but there were no specific measures in place at the federal to ensure online access for all. The study by Esses et al^18^ illuminates how lack of policies to address the digital divide particularly impacted newcomers. Virtual learning and social distancing in schools had severely impacted children’s language acquisition, socialization, and integration. Similarly, Ruckert et al find that low-income, newcomers and Indigenous children faced challenges accessing online education due to the lack of access to technology. This meant the burden of home schooling was left to parents, most often mothers, who faced difficult choices between children’s educational needs and their own employment.^27^

 Focusing on those with care responsibilities for older adults, Stall et al^21^ analyse long-term care policies during the COVID-19 pandemic and their impact on the older adult residents and their family caregivers. The visitor policies varied across provinces but were consistent in restricting family support systems during lockdowns and outbreaks. The policies lacked flexibility and disregarded the evolving needs as the pandemic progressed. Furthermore, Stall et al argue, the policies failed to identify family caregivers as a distinct group who provide essential services for residents with dementia, such as feeding assistance and medical decision-making, beyond social reasons. These restrictions caused significant declines in residents’ functional and cognitive abilities, physical and mental health, responsive behaviors, and increased loneliness. The lack of transparency and communication regarding visitor policies caused major frustration among nursing home residents, their families, and friends.

 Two studies note how the initial lockdown and subsequent service disruptions increased the risk of violence for women and children. Smith et al^27^ demonstrate how lack of sustainable support for the violence response sector pre-pandemic meant that facilities did not have the resources and staff to meet needs during lockdown, limiting the effectiveness of emergency investments in shelters. They further note interruptions to childcare resulted in increased contact with partners with past histories of violence, as well as a rise in conflict with existing partners as new shared care agreements had to be negotiated. While provincial responses to increased violence included funding for virtual counseling services, those most vulnerable had inequitable access to technology and private space. The closing of community-based organizations interrupted the networks of support for women experiencing violence.

 During the initial months of the pandemic, the Katz et al study^29^ observed an overall trend of decreased reporting of suspected child abuse by school personnel (70.3%) and child protection workers (2.1%), and indicated that these groups could not effectively identify or report instances of abuse due to lockdowns or restrictions. On the other hand, there was a marked increase in the number of reports made by individual citizens (11.8%), parents (31%), and neighbors or family acquaintances (12.1%) showcasing increased community vigilance. While many services were shutdown, select social programs such as child protection services were excluded and saw a 4.8% increase in reporting by their department in the first year of the pandemic. Most of the department’s work involved developing routine activities with the child and family who were now at home consistently. Social workers struggled to increase connection with the families and the ability to conduct home assessments was restricted due to local guidelines.

 Spence et al^22^ assess the country’s pandemic response in relation to the needs of Indigenous communities. The federal government dedicated $515.2 million in funding to support Indigenous communities and enhanced access to further universal funds. Most dedicated funds were allocated to service providers and organizations directly providing support and aid for Indigenous communities, both on- and off-reserve. This support was bolstered by an additional $339.1 million for Indigenous businesses and transportation. However, Spence et al argue that the division of support among several communities was insufficient to meet community needs. Limited assistance was offered to off-reserve Indigenous Peoples, even though 55.8% of registered First Nations live off-reserve. Their findings underscored the diversity in socioeconomic, cultural, and health vulnerabilities among Indigenous communities throughout the nation, and how a pan-Indigenous approach impeded the effectiveness of such policy intervention.

###  Recommendations From the Literature

 Recognizing that most of the secondary effects of the pandemic reflect pre-existing structural inequities, many of the recommendations in the literature suggests first addressing these. For example:

- Economic relief programs such as Universal Basic Income or Targeted Basic Income in combination with employment insurance could help balance power dynamics between workers from marginalized groups and employers, providing workers the ability to voice their concerns regarding occupational health and safety, including during public health emergencies.^20^
- A longer, flexible model of non-consecutive parental leave could prove responsive to unpredictable demands of childcare.^23^
- Greater federal and provincial funding and support for immigration-serving agencies, and targeted policies and public guidelines to address the vulnerabilities that newcomers face.^18^
- The development and funding of a national plan to include digital literacy in all areas of settlement programs, from language programs to first language supports.^18^

A few recommendations also focus specifically on pandemic response policies:

- Additional public investiture in child protection services, and improved strategies for children to reach out to when their safety is threatened during lockdowns, with focused support for disadvantaged youths and children who may suffer from repeated risks.^29^
- More explicit gender-targeted policy measures to address setbacks to gender equality.^27,31^
- The implementation of flexible and compassionate policies supporting family caregivers of older people during emergencies, recognizing that blanket policies may not be sufficient for all.^21^
- Policies that facilitate family caregivers taking on informal roles to enable residents to receive culturally safe and appropriate care, especially for 2SLGBTQ+ and Indigenous residents and/or those with language barriers.^21^

In addition, a group of recommendations focused on how decision-makers might engage with priority populations.

- Policy and decision makers should engage with racialized and marginalized communities as partners and co-owners in desegrated data collection to ensure appropriate use and identify inequities.^30^
- Consultation with migrants in developing socio-economic response strategies as well as how to mitigate barriers in accessing health, social security services and information into policy responses.^20^

## Discussion

 The COVID-19 pandemic motivated an exceptional policy response that dramatically affected everyday life across all segments of Canadian society. Substantial research has demonstrated that these effects were disproportionately experienced by priority populations. Moving beyond documenting these inequities, this review has focused on analyses of the policies that contributed to them, in order to inform more effective equity-based pandemic response going forward. The literature included, particularly highlights how policy responses either exacerbated old or created new inequities among migrants, women, children, and those of lower socio-economic status. To a lesser extent, it documents how policies failed to address the unique experiences of 2SLGTBQ+ people, older adults, those living with disabilities and Indigenous Peoples. The limited number of policy analysis studies to date suggests there is still much scope for research recognizing policies as structural determinant of health inequities in Canada, including that which takes an intersectional approach.

 A review of strategies adopted by 15 countries to mitigate the unequal effects of the pandemic found similar results to this review, in terms of states implementing a wide range of policies but which were insufficient to address underlying and exacerbated inequities.^33^ Countries with similar commitments to gender-equality, such as Iceland, were also unable to avoid the unequal effects of the pandemic born by women.^34^ Such global trends point to the influence of global political and economic determinants of pandemic response policy, a topic requiring further research.^35^ Revised conceptualizations of the social determinants of health framework in the context of COVID-19 may help explain these global trends by providing a more nuanced categorization of socioeconomic and political context, including oppressive systems such as patriarchy, and consideration of axes of inequity.^36^

 Bringing together research analyzing Canada’s policy response advances understanding of where claims to implement an equity-based response, or consider the needs of those made most vulnerable, fell short and why. While the studies focused on effects across different priority populations and policy sectors several common themes are apparent. First, research agrees that as opposed to creating new inequities, the response to COVID-19 exacerbated pre-existing inequities caused by inadequate labour protections and recognition of the importance of the care economy, as well as persistent disregard to the rights of priority populations such as migrants and people living with disabilities. Therefore, pandemic preparedness must include addressing these fault lines in social and economic security. Second, findings indicate the general support programs, while having some positive effects, were inadequate to meet the specific needs of priority populations. Pandemic response policies need to be tailored to address inequities experienced by migrant labourers, single mothers, and people living with disabilities – to name a few. Thirdly, numerous studies point out how lack of clarity around policy responses led to confusion or uncertainty around eligibility and how to access or utilize specific services or programs, leading to further barriers. This was particularly experienced by people with low income, immigrants, and women facing gender-based violence, and had severe ramifications for their health and safety.

 Finally, numerous studies focus on how the policy response exacerbated gender inequality, noting that these effects were pronounced despite Canada’s commitment to GBA Plus. The impacts of the pandemic were gendered in many facets with a exponential increase in the burden of care and gender-based violence, which had a disproportionate effect on women.^27,31^ The studies by Doucet et al^23^ and Katz et al^29^ further elaborated on the social and security impacts of the pandemic on children and their parents. The marked reduction in child abuse reporting and limitations by authorities conducting home visits put children at risk. Furthermore, the additional burden of childcare and limited access to benefits, resources and supports had adverse financial and risky implications for these families. This was found to be particularly relevant for 2SLGBTQ+, single parent, and racialized families that are historically of lower socio-economic status.^30^ Such analysis suggests that GBA+ needs to be proactively integrated into Canada’s pandemic preparedness and response planning, with greater consideration of the intersecting inequities that interact with gender to determine health, social and economic outcomes. While there have been calls for intersectional and equity-based pandemic responses, there is a dearth of analysis to inform such policy development.^37^ This review contributes to filling this gap, but much further research and analysis is needed.

 This review found just 14 studies analyzing Canada’s policy response to the pandemic from an equity perspective. Not surprisingly, there are many gaps in this literature. The existing research is largely focused on policy analysis related to a narrow range of priority populations with only one study each linking policy analysis and the effects on Indigenous Peoples, children, and older adults. Other studies only touched on policy effects on other priority populations such as adolescents, sexual and gender minorities, and people with disabilities. This review has also underscored the lack of equity- or intersectional-based perspectives and existing methodologies in disaggregated data collection, notably for racialized and Indigenous peoples, older adults, and people with disabilities.^20,23,30^

## Conclusion

 While limited, research analyzing Canada’s pandemic policies makes it clear that pandemic preparedness and response involves recognizing the importance of investing in healthcare, social infrastructure, and economic supports as a key component of pandemic preparedness. This includes developing a robust social and economic safety net that can support individuals and communities during times of crisis. However, it is important to recognize that pandemic response cannot be a one-size-fits-all approach. Different populations have unique needs and experiences that require tailored solutions. The specific needs of priority populations need to be considered by policy makers, including the compounding vulnerabilities for those who stand at the intersection of marginalized social identities. Canada is in a unique position with its commitment to GBA+ analyses and acknowledged feminist response, but results suggest it still struggles to move beyond bureaucratic barriers and address the implementation gaps for an equitable pandemic response. With reflexive humility and willingness to engage with members from priority populations, Canada can be informed and prepared to address the needs of priority populations during crises.

## Acknowledgements

 We would like to express thank you to team members who supported the conceptualization of the paper including Neda Zolfaghari (Simon Fraser University), Dawn Hoogeveen (Simon Fraser University), Miga Chultam (WAGE), and Samantha Ghanem (PHAC) for their invaluable insights and thoughtful suggestions, which greatly improved the quality of this paper.

## Ethical issues

 Not applicable.

## Competing interests

 Authors declare that they have no competing interests.

## Funding

 This work was supported by the Canadian Institutes of Health Research under grant number 486835.

## Supplementary files

Supplementary file 1. Scientific Database Search Strategy for Equity Focused Analysis of Canada’s COVID-19 Response Literature.
