# Supplementary material for: Equity Lens on Canada’s COVID-19 Response: Review of the Literature
Source: Int J Health Policy Manag. 2024 May 7;13:8132. doi: 10.34172/ijhpm.2024.8132 (PMC11270619; doi:10.34172/ijhpm.2024.8132)
Supplement: Supplementary file 1 — Scientific Database Search Strategy for Equity Focused Analysis of Canada’s COVID-19 Response Literature. [file ijhpm-13-8132-s001.pdf]

Article title: Equity Lens on Canada's COVID-19 Response: Review of the Literature

Journal name: International Journal of Health Policy and Management (IJHPM)

Authors' information: Muhammad Haaris Tiwana[1]*, Julia Smith1, Megan Kirby[2], Simran Purewal[1]

[1]Faculty of Health Sciences, Simon Fraser University, Burnaby, BC, Canada.

[2]Department of History, Faculty of Liberal Arts & Professional Studies, York University, Toronto, ON, Canada.

***Correspondence to:** Muhammad Haaris Tiwana; Email: mhtiwana@sfu.ca

Citation: Tiwana MH, Smith J, Kirby M, Purewal S. Equity lens on Canada's COVID-19 response: review of the literature. Int J Health Policy Manag. 2024;13:8132. doi:10.34172/ijhpm.2024.8132

Supplementary file 1. Scientific Database Search Strategy for Equity Focused Analysis of Canada's COVID-19 Response Literature

Pubmed

1. Exp Canada/
2. Exp Covid-19.mp OR sars-cov-2.mp OR corona virus.mp OR coronavirus.mp
3. Exp Pandemic/
4. Exp disease outbreak/
5. 3 AND 4
6. Exp policy/
7. healthcare policy.mp OR healthcare planning OR health service* OR public health systems
8. 6 AND 7
9. 1 AND 2 AND 5
10. Exp inequit*
11. Exp inequalit*
12. 10 AND 11
13. Exp intersectional*
14. Exp marginaliz*
15. 8 AND 9 AND 12
16. 15 AND 13 AND 14
17. Limit 16 to (human and English Language and yr="2019-current"

Scopus

1. ALL (Sars OR coronavirus AND disease OR severe AND acute AND respiratory AND syndrome OR sars AND virus OR sars-cov OR sars-related AND coronavirus OR sudden AND acute AND respiratory AND syndrome) AND (LIMIT-TO (LANGUAGE , "English")
2. ALL (inequity OR inequities)
3. ALL (Canada OR Canadian)
4. #1 AND #2 AND #3
5. ALL (policy OR Health policy OR healthcare policy OR health service OR public health systems) AND (LIMIT-TO (LANGUAGE , "English")
6. ALL (vulnerable OR marginalized)
7. #5 AND #6
8. #4 AND #7

Web of Science

1. TS=(covid-19 OR sars-cov-2 OR "corona virus" OR coronavirus) OR AK=(covid-19 OR sars-cov-2 OR "corona virus" OR coronavirus) OR KP=(covid-19 OR sars-cov-2 OR "corona virus" OR coronavirus)
2. TS=("inequity" OR inequalities)
3. TS=(policy OR healthcare policy) OR AK=(policy OR healthcare policy) OR KP=(policy OR healthcare policy)
4. TS=(Canada)
5. #1 AND 2 AND 3 AND 4
6. TS=("vulnerable" OR marginalized)
7. #5 AND #6
8. #7 AND LANGUAGE: (English) Timespan=2019-current

CINAHL

1. Covid-19 OR sars-covs-2 OR “corona virus” OR coronavirus
2. Canada OR Canad*
3. MH policy
4. “healthcare policy” OR “healthcare planning” OR “health service” OR “public health systems”
5. S3 OR S4
6. S1 AND S2 AND S5
7. MH inequity
8. MH inequality
9. MH marginalized.
10. S7 AND S8 AND S9
11. S6 AND S10

JSTOR

All fields (“Covid-19” OR “Sars” OR “Coronavirus” AND “Canada” AND “Policy” OR “Healthcare Policy” OR “Health Service” OR “public health system” AND “Inequity” AND “Inequality” AND “Marginalized” AND “vulnerable”).
